# Ensuring Privacy and Confidentiality in Social Work Through Intentional Omissions of Information in Client Information Systems: a Qualitative Study of Available and Non-available Data

**DOI:** 10.1007/s44206-022-00029-9

**Published:** 2022-11-19

**Authors:** Diana Schneider

**Affiliations:** CC Emerging Technologies, Fraunhofer Institute for Systems and Innovation Research ISI, Karlsruhe, Germany

**Keywords:** Privacy, Digital case records, Client information systems, Intended omissions of information, Bias

## Abstract

There is intensive debate about the use of AI-based systems in social work, although the degree of digitalization is low in many jurisdictions. In this article, the findings of research about the process of case recording in client information systems for social work are presented. Between January and June 2020, 20 guideline-based interviews were conducted with experts working for funding agencies or service providers. A significant finding was that there are intentional omissions of information within digital client records, despite being relevant for further case processing. This finding and the reasons for omission are highly relevant to the current debate on AI, because it extends consideration beyond the ethics of design to include the ethics of usage.

## Introduction

The increasing ubiquity of information and communication technology (ICT) in the social welfare sector has led to intensive debate about the development and use of AI-based systems. This debate has become increasingly focused on applications that regulate human interactions and activities. These include applications that affect decisions about people, for example, by predicting the probability of recidivism of delinquency, such as the COMPAS risk assessment software (Brennan et al., 2009; Larson et al., 2016; Vaccaro, 2019), or by scoring and categorizing job seekers into categories in order “to increase the efficiency of its counseling process and the effectiveness of active labor market programs” (Allhutter et al., 2020) such as the AMS profiling system of the Public Employment Service Austria (Arbeitsmarktservice, AMS) (cf. Holl et al., 2018). The Automating Society Report 2020 lists many other algorithmic decision-making (and decision support) systems in Europe that are intended to support and improve the lives of service recipients. Algorithmic decision-making systems “affect almost all kinds of human activities, and, most notably, the distribution of services to millions of European citizens—and their access to their rights” (Chiusi et al., 2020, p. 6) and consequently could pose a threat to existing forms of democracy. Much of the debate has focused on automated decision-making using predictive analytics and both supervised and unsupervised machine learning. Algorithmic systems have been criticized because of serious errors in their design and implementation. Specific criticisms have been the lack of transparency of algorithmic systems (the so-called “Black Box” (Gillingham, 2016; Wachter et al., 2017)); biased or missing data; the unquestioned assumptions of developer and unintended consequences (Chiusi et al., 2020; Crawford, 2013; Datta et al., 2015; Eubanks, 2018; Spielkamp, 2019; Zweig et al., 2018). The use of such systems brings with it the risk of errors of commission and omission using algorithmic decision support systems (DSSs) (Neri et al., 2020). Users may be influenced in their decision-making by the algorithmic system, but they might also manipulate systems through inputs designed to achieve certain outcomes (Kolleck & Orwat, 2020).

Algorithmic systems are, of course, also reliant on the data used to develop them. This data may be biased in ways that lead to discrimination, and its use raises questions about privacy and data protection. A fundamental question is the identification of exactly what data an algorithm is designed to use, and what data it can actually access. A preceding question also arises about the nature of such data and the process by which it was generated. The research described here aimed to address this question by exploring the processes by which data is recorded by social workers and decisions are made about what is recorded as data, what is not recorded and the legal, ethical, and professional considerations that arise.^1^ The findings suggest that such scrutiny is important to the assessment of the utility of data for the development and use of algorithmic systems. This is of particular pertinence to other professions, such as IT professionals, who have to evaluate the quality of data contained in client information systems (CIS) used in social work.

## The Debate About Client Information Systems in Social Work

It is the intensive and high-level discussion about artificial intelligence (AI) that in some cases neglects the fact that the degree of digitalization is low in many jurisdictions. Professions such as medicine (e.g., general practitioners), nursing, or social work also have to deal with changing work domains because of digitization processes—including both information and communication technologies (ICT) and AI (Webb, 2003). However, discussions in these fields are less concerned with automated decision-making than with supporting decision-making processes and how far processes can and should be digitized (Bastian, 2014; Devlieghere et al., 2022; Kreidenweis, 2018). Case recording in terms of what gets recorded has also been the subject of debate in social work. This debate became more focused with the advent of CIS as decisions about what gets recorded, when, how, and by whom were being made by CIS designers and managers with no frontline experience. Debate has also now turned to what we do with all this data, and data science has introduced the possibility of predictive analytics in all its forms.

Much of the research and commentary about CIS in social work rests on the assumption that “accurate recording and inter-professional information-sharing can enhance care in health and social services” (Cairns et al., 2018, p. 350; cf. Devlieghere et al., 2022, p. 2). A further assumption is that case records should contain all information which is essential to the process of making decisions and taking actions about a case. As such, case recording has been considered as part of the professionalization of social work (Gillingham, 2020; Merchel, 2004). Case records can also be used to demonstrate accountability and to evaluate and plan services (Heiner, 2011; Merchel, 2005; Merchel & Tenhaken, 2015). They may also be shaped by the criteria set (usually by governments at various levels) for eligibility for services. Hence, the creation of case records is influenced by the need to meet and anticipate multiple possible uses, some of which may exist in the present and some in the future (cf. Berg, 1996, p. 518).

Social welfare agencies may have extensive procedures about case recording, and there may also be established routines about what should be recorded (Reichmann, 2016) but, ultimately, it is left to the discretion of individual professionals to decide what to include and what to omit in case records (cf. Berg, 1996; Gillingham, 2015; Taylor, 2008). This is an example of what Lipsky refers to as “street level bureaucracy,” in which social workers act as the interface between state policy and individuals requiring a service. Social workers are expected to match up sometimes general and quite vague social policy with the complex needs of individuals. As Lipsky describes, this may be achieved by social workers “by developing routines of practice and psychologically simplifying their clientele and environment in ways that strongly influence the outcomes of their efforts” (Lipsky, 2010 [1980], p. xii).

Since the 1970s, there is also critical debate about the constructive character of case recordings. Previous studies highlight the construction of a case during recording (Berg, 1996; Garfinkel, 1967; Taylor, 2008) and emphasize that this case construction is used to legitimize professional actions (Ames, 1999; Gillingham, 2020). According to Merchel and Tenhaken (2015, p. 172, own translation), records provides “information about the views and interpretations of the person recording, about their selective perceptions, their categories, and their theoretical outlook.” Even today, the question of how to evaluate the relationship between records and reality is still being discussed (Gillingham, 2015, 2020; Merchel, 2004). Gillingham (2015, p. 1600), for example, speaks of “large gaps” to emphasize the difference between the two. Due to their stigmatizing effects (Heiner, 2011; Merchel, 2004; Webb, 2003), records “should treat a client fairly by demonstrating respect for them” (Cairns et al., 2018, p. 360).

If the intention is to use case records as “data” for predictive analyses, it is important to be aware that CIS might have a significant influence on how case recording is conducted (cf. Aas, 2004; Parton, 2006). Scholars report deliberate manipulations and dummy entries (Büchner, 2020; Gillingham & Humphreys, 2010), deliberation and interpretative “making […] fit” of given categories (Bastian & Schrödter, 2014, p. 280, own translation; Shiller & Strydom, 2018), redaction, ignoring, or deliberate expansion of certain defaults (Ackermann, 2021), among other things.

## Expectations With Regard to What Is Not to be Found in the CIS

The question of whether and, if so, under which circumstances there are omissions in digital records has been addressed in some research (cf. Gillingham, 2015, 2020); albeit often marginally. Non-available data are both unknown data and data that are not entered into CIS. Some omissions are the result of limitations in both the hardware and software used in CIS (cf. Gillingham & Graham, 2016; Huuskonen & Vakkari, 2013, 2015; Merchel & Tenhaken, 2015). Furthermore, restrictions due to organizational framework conditions (e.g., lack of resources, high workload) and personal factors (e.g., lack of understanding of the importance of recording (Merchel & Tenhaken, 2015)) can influence the quality and scope of recording.

However, few studies are also devoted to the phenomenon of deliberate omissions in digital case records (Cairns et al., 2018; Huuskonen & Vakkari, 2015; Polychronis, 2020; Zanchetta et al., 2015). Another reason for omissions in case records is that service users expect that the information they impart to social workers is bound by confidentiality (Cairns et al., 2018; Polychronis, 2020; Stablein et al., 2018). Indeed, research has found that “[e]thical tensions can arise for health social workers [that means, social worker who are part of an multidisciplinary health care team, DS] between comprehensive recording and protecting client privacy by limiting access to privileged information obtained in the context of a social work encounter” (Cairns et al., 2018, p. 348). Stablein et al. (2018) report “concealing information and utilizing sets of personal and collective codes designed to alert providers or teams of providers to confidential information within a patient’s record.”

Confidentiality in professional social work practice is one of the core values and a “cornerstone of professional social work relationships” (Canadian Association of Social Workers (CASW), 2005, p. 7). According to the German Criminal Code (Strafgesetzbuch, StGB), the duty of confidentiality applies, among others, to state-recognized social workers or state-recognized social pedagogues (cf. Section 203, subsection 1 StGB: violation of private secrets). For professional work, CASW (2005, p. 7) defines that “[s]ocial workers demonstrate respect for the trust and confidence placed in them by clients, communities and other professionals by protecting the privacy of client information and respecting the client´s right to control when or whether this information will be shared with third parties.” In this sense, the data collected in the context of case recording usually belong to special categories of personal data that are considered to have a high level of protection (cf. Section 9, subsection 1 of the European General Data Protection Regulation, GDPR; Pudelko & Richter, 2020). Accordingly, the processing of this data^2^ in order to decide, for example, on social services, must comply with applicable data protection and data security laws and regulations. At the same time, however, data protection itself plays an ambivalent and ambiguous role in social work practice in Germany (cf. Pudelko & Richter, 2020, p. 414). According to Pudelko and Richter (2020), although there is an awareness in German social work of the need to implement legal frameworks to protect personal data, the challenge lies in how exactly to implement these requirements in daily practice. One of the biggest challenges is the European General Data Protection Regulation (GDPR) itself. The application of the GDPR addresses people who are assumed to be capable of informed consent, but this assumption cannot be taken for granted everywhere in the field of social work. Therefore, social workers attempt to compensate for this discrepancy through their so-called double mandate: they not only see themselves as being obligated to the state as a funding agency and principal, but also orient themselves towards the needs and requirements of their clients (cf. Hoffmann, 2020, p. 47). In doing so, ensuring the privacy of vulnerable people could play a decisive role in the whole process of social care service planning, including their digital case recording.

## Study Design and Results

The current case recording practice within social organizations was analyzed as part of the interdisciplinary project MAEWIN^3^ (2018–2021). The aim of the MAEWIN project is to explore the possibility of using automated procedures for text and data analysis to generate evidence-based recommendations for actions in the field of social care services. In addition to assessing what information is available for this process in the database, this research has also aimed to develop an understanding of how it was created. The field of study is the provision of services to people with disabilities, with the aim of maximizing both their self-determination and their participation in society.

### Methodology

In contrast to the international discourse in social work, there are no DSS used in Germany that could be investigated for this purpose. The aim of the first stage of MAEWIN study therefore was to gain an overview of existing and non-existing data points within the CIS and identify those “large gaps” (Gillingham, 2015, p. 1600) that are highlighted in the literature to underline the difference between the recorded content and the actual information available. The prevailing opinion among scholars is that these gaps ensure that the (digitally available) data “is insufficient information to gain any real understanding of either the service user or the response provided” (Gillingham, 2015, p. 1600). Having identified existing gaps for the specific context of social care service planning for people with disabilities, the next step is working out how data might be used to develop DSS and to consider more concretely the potential use of algorithmic systems for decision-making. First considerations and tentative conclusions on this have already been published in Schneider et al. (2022).

### Data Collection, Participants, and Data Analysis

Between between January and June 2020, twenty guideline-based interviews with experts working for funding agencies of integration assistance (German: *überörtlichen Träger der Sozialhilfe und der Eingliederungshilfe)* or social service providers (purposive sampling) were conducted. At the time of the interview, respondents worked in either North Rhine-Westphalia or Berlin and were between 29 and 63 years old. Interviews were recorded and lasted on average 122 min. The recordings were transcribed and then by using qualitative content analysis, according to Kuckartz (2014) via MAXQDA (version 2020).

In the following analysis, the following questions from the interview guide are focused on:

- Which data are/ should be recorded?
- Which data are/should not be recorded?
- Why are data recorded?

The following presentation of these aspects by the interviewees is summarized in a condensed form. Only in isolated cases, quotes from the interviews are mentioned to better illustrate some aspects. In many passages of the interview, the respondents refer to the specific context of planning social care services for people with disabilities in order to highlight its specificities. This contextual embedding is maintained in the presentation of the results.

### Findings I: Available Data

Because social care service planning is subject to the legal regulations of integration assistance in Germany, the necessary criteria for an individual and function-related need assessment can be inferred from the legal text (cf. Section 13, subsection 2 SGB IX new version). This includes recording on whether a disability exists or is likely to occur, what effects the disability has on the social participation of the service user, and which goals and aims of participation are to be achieved with social care services. It is also necessary to note which services are to be used to achieve the goals of the person with disabilities. In addition to recording relating closely to the application for social care services, further case records can be found within funding agencies and social service providers. Most respondents report, keeping a more or less extensive electronic file on their clients. People who are employed by social service provider also record daily events of the clients within the framework of their professional work (see Table 1). The majority of the available data is recorded in semi-structured or unstructured data (text files/free text).

**Table 1:** Selection of recording available in the social care service planning and their data type (own representation)

**Recording aim**		**Data type and content**	
		**Structured data**	**Semi-structured or unstructured data (especially text files/free text)**
**… whether a disability exists or is likely to occur**		• ICD code • Medical diagnoses	• Medical reports • Description of restrictions to participation (orientated towards ICF code) • Documents that prove that a participation restriction is basically (e.g., school or work reports, certificates)
**… what effects the disability has on the social participation**		• If applicable: professional categories or codes (e.g., Metzler code; not necessary semantically coded data) • If applicable: professional statements through scale/ scores	• Description of restrictions to participation (orientated towards ICF code) • (Self-)description of a person’s abilities, skills, resources, and preferences • (Self-)descriptions on the (non-)domestic problems, barriers, and challenges affecting daily life • Professional statements through report/description
**… which goals and aims of participation are to be achieved with social care services**			• Definition and prioritization of areas for support
**… which social care services, in the context of a forecast for the achievement of the goals, are likely to be successful**			• Plan, manage, and evaluate actions such as measures/agreements on the content of what is to be implemented in the future incl. time planning
**… personal data**		• Name • Age • Address • Data about the personal and factual circumstances	• Data about the personal and factual circumstances
**… further data depending on the organization and case**	Funding agencies	Among others: • Statistics on service users • Statistics on social services provided, i.e., social activities approved • Log of the processing status of an application	Among others: • Background information/ back-up recording for situations that may require a future reconstruction of action • Professional reflections/statements, interpretations, perceptions, or theoretical outlook
	Service provider	Among others: • Vital data • Medication lists • Proof of activities by clicking off measures	Among others: • Proof of activities by writing text • Accident logs and other logs • Daily events and/or new information with implicit and/or explicit impact on the mandate to act • Background information/back-up recording for situations that may require a future reconstruction of action • Professional reflections/statements, interpretations, perceptions, or theoretical outlook

Assessing the relevance of information is a difficult undertaking. Respondents had different strategies for determining relevance; for example, some mentioned that information is relevant, if it has an implicit and/or explicit impact on the social workers’ mandate to act (cf. purpose limitation, Sect. 5, subsection 1, number b GDPR). Such information can be, for example, new wishes and goals of the service user, or a change in personal circumstances. However, the relevance of these changes is a matter to be decided by the case worker:

“When a client falls in love, it’s a very, very personal story. When he confides in me: I have fallen in love. Then this is to be recorded if a need for support arises from it. But if that is not relevant—although, with this topic, it has relevance [to work, DS] in many places.”

In some cases, intimate information that the service user does not want recorded or passed on to third parties may be disclosed. In this instance, professionals must make decisions about whether such information is recorded and whether it is passed on to third parties, despite the wishes of the service user:

“And if clients say you don’t write that down. (...) Then I have to weigh it up - THEN I have to decide: Is it important that I write it down anyway? Then of course I have to make it transparent and say: Listen, [...] we are obliged [to record, DS].”

“That sounds quite nasty now, because it is actually an abuse of trust on the part of the caregiver in relation to the person with a disability. When they [the caregivers, DS] tell us things that the service user does not want us to know. However, these statements are so important, because otherwise we do not understand certain things. Because these are the unspoken gaps.”

The need for privacy can, therefore, be superseded by the perceived need to record and share relevant information. Relevance can also be ascribed to professional observations and understandings, but they may not fit into the prescribed spaces in a CIS. Case workers find ways to circumvent the restrictions of the CIS:

“Yes, we have this standard protocol, which everyone [...] fills out. They all look the same. If there was a conversation about it [about participation restrictions, DS], there is always another blank sheet. Because this protocol does not allow you to record a conversation or record things [for yourself, DS]. So you just have to be inventive.”

The assessment of relevance can therefore be made for different reasons and, in case of doubt, is at the discretion of the individual professional.

Participants also raised the problem that some information recorded in case files can be derogatory about service users or could be interpreted as derogatory. For example, some respondents reported that records included derogatory remarks about the clients (e.g., “spiteful, slanderous remarks”, German: *Hetze*), or that the judgments of some of their colleagues were “mean, wrong, unprofessional, foul-mouthed.” Some respondents concluded that records should be free of personal assessments:

“Yes, I think that emotionality or stories like that should be left out of the recording. Because I think everyone feels that differently and sometimes the situation is distorted as a result. Instead, it should be more about the fact that a third party, under certain circumstances, who is doing a substitution or taking over things, is informed without being pushed in a certain direction. Like that. I think that’s important. And that it is formulated in such a way that the client can look in at any time without being stigmatised in any way. [...] But that facts [rather than hypotheses, DS] are recorded.”

“Personal evaluations, especially insults, have no place in the file.”

A few respondents point out that respectful recording is also challenging for professionals, particularly when the meaning of certain words is ambiguous. Information may thus be quite vague and open to misinterpretation. For some respondents, there is a realistic risk that other professionals (e.g., their own colleagues) or third parties could misinterpret such appreciative descriptions:

“Professor Metzler has the word *support*, doesn’t she? This word *support*. I can classify it both under B, as help, qualitative need for help, and under D. It is a neutral word, a friendly word. The funding agencies of integration assistance always classifies it as B, because there is less money. We classify it as D, for example. And then I say: guys, never use the word *support* singularly, because there is scope for interpretation. Either you describe the need for help in such a way that it can be assigned to B. If that’s not the case, then you have to describe the need for help in such a way that there is no more scope for interpretation.”

### Finding II: Non-available Data

Respondents first addressed factors that are mentioned in the literature, such as the limitations of hardware (e.g., lack of mobile devices for adding to case records while away from the office) and software (e.g., limited space to record), as well as the organizational conditions (e.g., high workload). Participants also mentioned a number of factors that may be present in caseworkers, such as a lack of understanding of the importance of case recording, uncertainty about how to use software, laziness, forgetfulness, and a lack of literacy.

“The colleagues say they don’t have time to record. [...] They would like to do it, but they don’t have the time or the technical equipment. [...] So there is only one PC, three people have to record at the same time towards the end of the shift. There is a lack of resources. [...] For some [the recording] is an annoying evil. Who don’t feel like reflecting on their work or writing it down. Who may find it difficult to write. That is a big problem. Many are also not necessarily familiar with the German language or are ashamed if they have spelling problems.”

Some participants pointed out that a significant deficit in case files is the service user perspective. This omission arises in part because of the limitations of the assessment tool but may also be because of the limited communication skills of the service user. For example, one respondent mentioned the “power imbalance” that exists in the process of case recording: case records are generally created and held exclusively by social welfare agencies rather than service users. An exception to this general rule occurs with the inclusion in case records of “deputy statements.” These may be prepared for people with limited communication skills in order that their wishes and opinions can be included in case reviews. The lack of client perspective in the records is also expressed in the desire of fewer respondents for participatory recording:

“I think it would be good to be able to keep the records more flexible in the sense that it is not tied to the office. So, I think it would be better - this is also being discussed at the moment in our organisation - to have portable notebooks, so that I can have them with me and, for example, also record with clients, together. That you sit down briefly at the end of a contact and look: Okay, what was important today? What do we want to record? So you can involve them [the clients, DS].”

Overall, participants, for all the reasons mentioned above, considered that case files are incomplete, a partial version of “reality” and require interpretation. Interpreting the meaning of the case recorder, though, may not be easy or precise and so there is a level of uncertainty in using case files in day to day practice. As one participant described, this uncertainty has to be “endured” by social workers and is considered to be part of the professional role of social workers (see Schon’s, 1984) depiction of professional practice in the “swampy lowlands”). This perception is expressed particularly succinctly in the following quote from a respondent:

“Paper is patient. You can imagine a lot, but you can also imagine a lot of wrong things.”

Finally yet importantly, two participants were of the opinion that there should be nothing that should not be recorded. One participant spoke about the need for a “positive fail culture” within social welfare agencies.

“So, I think it would be wrong not to record that it also fails once. I also think it’s right that you record that you might also be wrong with your assessment.”

Almost half of the participants reported that they do not record certain topics at all or at least not in detail. In addition to traumas or experiences of violence, respondents mentioned past life events that have no impact on the current situation (e.g., imprisonment), relationship problems including sexual relationships (e.g., partnership problems, venereal diseases, or frequency of sexual contacts), a client’s worldview and psychological problems that require therapy. Participants justified these omissions with reference to the very personal and potentially sensitive nature of some information. One respondent summed it up as follows:

“There are definitely stories that do not have to be recorded anywhere.”

Although respondents acknowledged the needs for high levels of confidentiality, they were also aware that this seems to be not possible in the context of a social welfare agency. In five interviews, the interviewees point out that colleagues could see the data recorded in the CIS.

“We can look into the [CIS, DS] at any time. [...] This is used by EVERYONE of us and there is no other way. Otherwise we wouldn’t be able to work at all.”

“Everything we record is accessible to all. So “accessible to all” means: In our system, everyone who has an authorisation to look into these files can see that.”

Participants were also aware that information recorded in a case file exists permanently. This ubiquitous access led some respondents to reflect on the process of recording and its consequences.

“The question is: For example, would I record what happened [in this case] in such a protocol? There is a POTENTIAL danger; it could be that something like this could happen again. Do I have to record that or is that ethically wrong? Because it’s something that happened a long time ago [and doesn’t currently have a impact on the work with the client, DS]. So, do I maybe not need to write that in my internal protocol? On the other hand, the question is: who reads the internal protocol? So, that’s quite less a question of [internal, DS] recording. [...] Which might really only be read by myself as a reminder. Who already knows it anyway. Or my colleague who does the same work as I do.”

Participants’ responses describe two methods in which they respond to ubiquitous access in the records. First, certain topics and information might not be recorded due to ubiquitous access to the CIS:

“But I would not record intimate or very private details of a person there [in the CIS, DS] because, of course, that is data that, even if it is protected, may be accessed somewhere [by someone, DS], and that is also stored and exists.”

Second, participants would only record as much as they deemed to be sufficient for the required work to be done. One respondent describes the limited recording as recording “on a meta-level.”

## Discussion

The results illustrate that the content recorded depends on the relevance to the case. Relevance is assessed by the individual professionals. Thus, on the one hand, recordings and data transfer to third parties may occur even though the clients refuse this. On the other hand, information may be withheld (intentional omissions) or only recorded at a meta-level in the CIS. The reasons given by the participants may be understandable and plausible for members of the professions; however, this could be a challenge for people who want to process this data in the context of algorithmic analyses. Therefore, the following section attempts to identify a mechanism for this behavior. For this purpose, I draw on Rössler (2001) theory of privacy,^4^ which links informational self-determination with the relationship level of individuals and extend this theory in light of the above findings.

### Mechanism of How the Data Ends Up in the CIS

According to Rössler (2001), informational privacy is not only a yardstick for distinguishing between public and private relationships, but it is also necessary in a functional sense in order to be able to speak of a private relationship at all. Based on the relational theory of privacy, according to Fried (1968) and Rachels (1975), she links the concept of informational privacy to “the idea that privacy is a function of *relations* between individuals” (Roessler & Mokrosinska, 2013, p. 774, emphasis in original). Therefore, the decision regarding what information to share with others is not only subject to the personal discretion of the individual person, but also based on mechanisms “constituted and governed by standards external to and exceeding individual control” (Roessler & Mokrosinska, 2013, p. 775). In the context of planning social care services for people with disabilities, for example, it is necessary to know whether a disability exists; and if so, what effects the disability has on the participation of the service user. Within a professional setting, this information (and further information) has to be shared with members of funding agencies as well as of service providers. As Rössler points out, the mere sharing of intimate information does not mean that the relationship between the actors involved must necessarily be intimate as well. For example, the relationship with a professional (e.g., a psychoanalyst) or a stranger (e.g., a stranger on the train with whom one has a private conversation) is not intimate despite the sharing of intimate information (cf. Rössler, 2001, p. 237). It is nonetheless common for strangers outside of this professional setting to be unaware of these individual challenges or the resulting social care services. Interaction between strangers is normatively regulated by “respect for the privacy of others (indifference) and the retention of one’s own privacy (reserve)” (Roessler & Mokrosinska, 2013, p. 782).

In contrast to both the interaction between strangers and the professional setting, intimate relationships are characterized as spaces in which a person tries out “what a self-determined and authentic life could be, and determines which staging’s of self-presentation would be possible, desirable, authentic, and so forth” (Rössler, 2001, p. 235, own translation). According to Rössler, intimate relationships arise “in a *setting* characterized by friendly affection or love, by concern and consideration, and by a special form of interest” (Rössler, 2001, p. 237 f., emphasis in original, own translation). Intimate relationships are found “in a particular context of affective devotion, sympathetic attitudes, and commitments entered into between the persons involved” (Rössler, 2001, p. 238, own translation). Classically, the relationship to family, friends, and intimates is private; and these intimate relationships “need to have their privacy protected precisely because one of their functions involves retreating into a private world” (Roessler & Mokrosinska, 2013, p. 775). The relationship in social work is also “more dependent on an undisturbed, trusting relationship with the person seeking advice than in almost any other professional field” (Pudelko & Richter, 2020, p. 415, own translation). Therefore, the following consideration argues that professional relationships in the context of integration assistance can also be potentially one-sided intimate relationships in rare cases. This consideration contrasts with the idea of professional relationships as outlined by Rössler (2001) and Roessler and Mokrosinska (2013).

Therefore, it is helpful to visualize the particular setting within social work—for example, in the context of planning social care services for people with disabilities. In contrast to the usual setting between a professional (such as a psychotherapist) and clients that is clearly defined both spatially and temporally, social workers and clients meet in a variety of settings depending on the needs of their clients. In certain situations, it may be helpful or even necessary for the social worker/professional to come to the client’s home^5^ (Cairns et al., 2018, p. 356). In assisted living, the distinction between living and working spaces can no longer be determined clearly: what is the living space of one is the working space of the other. This means that some of these social care services take place not only metaphorically, but also actually in a space that is perceived as particularly worthy of protection: one’s own home (cf. Rössler, 2001). Even when these conversations take place on the premises of the organization, encounters can occur that, according to Rössler, take place in the symbolic space of intersubjective confrontation. The symbolic space of intersubjective confrontation belongs in the intimate setting because it must be understood as constitutive of the identity and autonomy of the person concerned (cf. Rössler, 2001, p. 239). According to Appelbaum (2002, p. 1811), this refers to a deontological argument for privacy:

“The ability to speak freely with another person about one’s innermost thoughts, fears, and passions is clearly dependent on the belief that one’s revelations will go no farther. Creating a space within which this sort of dialogue can occur is likely to facilitate the conscious exploration of alternative modes of thought and behavior on which truly autonomous functioning rests.”

In such situations, the relationship between professionals and clients can become almost informally friendly. Some social workers even see this as a quality feature of their work relationship (Zanchetta et al., 2015). In such settings, it can happen that the clients undertake such attempts at self-presentation that Rössler locates in the intimate setting. Statements in the interviews that something was told “in confidence” testify to this intimacy of the relationship. The practice of neutral recording also underlines the respect for clients. In this context, publicity does not begin only when information gets into the hands of third parties outside one’s own organization, but already at the time of *writing it down* in CIS, because “certain others” (Rössler, 2001) can view this private information about the person in question. This confirms that in certain cases the duty of confidentiality also seems to apply to one’s own colleagues. The knowledge gained in such (semi-)intimate settings must therefore be assessed in terms of its relevance for professional work and then, if necessary, transformed into the CIS (cf. Table 2).

**Table 2:** Perceived relationship level and its impact on the recording of information in CIS (Schneider, 2022, based on Rössler, 2001; own translation/minor additions)

	**Interpretation of the person recording: intimate relationship from the client’s perspective**	**Interpretation of the person recording: professional relationship from the client’s perspective**
**Intimate relationship from the point of view of the person recording**	• Intimate relationships • Privacy conflicts unlikely but possible • Records unlikely but possible	• Privacy conflicts unlikely but possible • Impact on records unclear
**Professional relationship from the point of view of the person recording**	• Conflicts of privacy • If applicable, transformation of knowledge: selectively omitting or recording on meta-level	• Professional relationships • Privacy conflicts unlikely but possible • “Normal” case recording (ideally neutral)

If the person recording receives the information in an intimate setting, recording of the contents is unlikely but possible. It can be assumed that professionals perceive existing records in such cases as, for example, “spite” (interview), gossip, or collusion (cf. Rössler, 2001). If the information provided turns out to be relevant for future professional work, recording becomes necessary. For the person recording, this can be a dilemma: On the one hand, control over relevant intimate information must be relinquished, because there is a certain obligation to disclose within the application procedure in order to be able to demonstrate plausible and comprehensible legitimacy for social care services to provide support for participation in social life. On the other hand, the information cannot be disclosed without jeopardizing the confidential relationship with the client and being guilty of collusion, as is evident in some of the quotes above.

As a potential solution, respondents seem to see themselves as so-called gatekeepers or protectors of the clients—not only with regard to strangers or third parties not involved in the planning process of social care services, but also with regard to their own colleagues and the CIS they use in their professional work. One consequence of this way of thinking and acting is, for example, that certain information is withheld to protect privacy and the confidential relationship with clients. Therefore, information is not even recorded electronically (selective omission), even though it is important for further case processing. Instead, important and relevant information is summarized and noted by professionals “on a meta-level” (interview). Cairns et al. (2018), Huuskonen and Vakkari (2015), and Zanchetta et al. (2015) also report both selectively omitting and recording on a meta-level, as mentioned earlier. The above results underlines the consideration that this behavior is not exclusively a response to shared electronic health records (EHRs), but also occurs when only members of one’s own organization have access to this data. Gillingham (2020) also pointed out that not all data about someone in the context of social services is available electronically, and therefore all one will ever see in the data is a partial version of what happens in a case.

Nonetheless, this behavior may become problematic if the available data in the CIS is to be used to decide on which social care services to deliver for participation. This is because the information that is sometimes withheld may be needed to describe the concrete needs of the client in a comprehensive way—only then can certain services be financed at all. Therefore, full omissions can be understood only as case-specific negotiation processes between all actors involved (namely at least, funding agencies, service providers, and the service user), and they are therefore probably rather the exception. In particular, the professionals working for funding agencies are challenged: Is the information included in the application for social services in written form sufficient for them to come to a judgment about the decision on social services? On the one hand, if it is possible, the privacy of service users will be protected and guaranteed. On the other hand, due to selective omissions of personal and sensitive client information in the CIS, there will be probably little evidence for an algorithmic (decision-making) system to comprehend specific client situations and reconstruct professional judgments.

### Limitations

The research is limited by a relatively small sample of social workers who work in a particular context in Germany, and so no generalizations can be made about the findings. Further research in different social work contexts is required to confirm, refute, and possibly build upon the findings. The research was also limited by its reliance on participants’ accounts of their practice. Further research could involve case file audits to determine, in more detail, what is and what is not included in case records and its relevance to the development of algorithmic tools.

## Conclusion

The aims of this study were to explore the kind of information that is recorded in a client information system (CIS) in day to day social work practice and to identify the information that will not be part of records. It was found that the data which is recorded may be stipulated through legislation and internal organizational procedures. There may also be technical limitations associated with both the hard and software used to record information about service users and delivery which prescribe and sometimes limit what can be recorded. At the level of individual practitioners, what is recorded and what is left out are subject to professional-specific ethics, such as privacy and confidentiality. A key finding was that, particularly in relation to personal and sensitive information, social workers have to decide whether to record information with reference to what they consider to be “relevant.” From the data, it appears that “relevance” is subjective and may differ between practitioners.

Two significant inferences can be drawn from the findings that relate to the development of algorithmic tool in social work. Firstly, bias does not begin with data mining or analysis, but is embedded in the decisions made by professions about what to record. AI applications are, therefore, characterized by a “double subjectivity” (Schneider & Seelmeyer, 2019, p. 119) (cf. Crawford, 2013; Gillingham & Graham, 2016; Zweig et al., 2018). An important step could therefore be to raise awareness among people who further process the recorded information about how data is recorded in practice.

Secondly, the findings show that the challenge of ensuring privacy arises not just in the process of AI data analysis but is integral to the process of case recording. This conclusion, familiar to experts as far back as Garfinkel, is especially elementary for individuals unfamiliar with the customs and record-keeping practices of social workers to understand where the limitations of algorithmic analyses based on case records might lie (cf. Gillingham, 2019). As the results illustrate, in some special cases, privacy may be the result of negotiation processes by professionals who choose not to enter information into a CIS. To identify such behaviors, it would be necessary to examine whether and to what extent ethical values such as privacy, data protection, and confidentiality are reflected in the records of specific cases. In this regard, it is not sufficient simply to refer to existing legal regulations and profession-specific guidelines or requirements (of recording), because these do not contain any information about the actual recording practice.

If we are aware of these two points, it becomes clear that in the current debate on AI, we should focus not only on the ethics of design but also (and once again) on the ethics of usage of AI. Understanding the record process in social and health care can be one element in getting a more realistic idea of what kind of analysis is not possible due to lack of information (e.g., due to the particular understanding of privacy in social work).

## Data Availability

The datasets generated and analyzed during the current study are not publicly available due the fact that they constitute an excerpt of research in progress. In justified individual cases, excerpts of the anonymized data can be made available; the interviews were conducted in German. A complete insight into the data is not possible due to the guaranteed anonymity of the study participants.

## Footnotes

1 This article is a substantially expanded and revised version as well as English translation of my findings first published in Schneider (2022).

2 According to the European General Data Protection Regulation (cf. Section 4, subsection 2), the processing of personal data includes “any operation or set of operations which is performed on personal data or on sets of personal data, whether or not by automated means, such as collection, recording, organisation, structuring, storage, adaptation or alteration, retrieval, consultation, use, disclosure by transmission, dissemination or otherwise making available, alignment or combination, restriction, erasure or destruction”.

3 The MAEWIN project (Decision support systems in welfare institutions) is part of the NRW Digital Society research programme. The project was extended to 2022 due to research disruptions imposed by COVID-19.

4 This book is also published in English in 2005, but here I refer to the German edition.

5 Under certain circumstances, a home visit can have a (strongly) controlling character (e.g., in assessing the housing situation in the context of determining child welfare risks). Although such home visits sometimes focus less on empowerment and more on controlling, it may remain, however, a setting that differs from encounters with professionals from other professional groups.

## Code Availability

Inspections of the coding data system supporting the results of this study is available in justified individual cases upon request from the corresponding author.
